# O.4.3-5 Accelerometer-measured sedentary behavior among school-aged children, adolescents, and adults in Finnish population-based studies

**DOI:** 10.1093/eurpub/ckad133.191

**Published:** 2023-09-11

**Authors:** Pauliina Husu, Henri Vähä-Ypyä, Kari Tokola, Harri Sievänen, Tommi Vasankari

**Affiliations:** The UKK Institute, Finland; The UKK Institute, Finland; The UKK Institute, Finland; The UKK Institute, Finland; The UKK Institute, Finland; Faculty of Medicine and Health Technology, Tampere University, Finland; pauliina.husu@ukkinstituutti.fi

## Abstract

**Purpose:**

Sedentary behavior (SB), mainly sitting, covers most of the waking hours in Western populations, and is found harmful to health and well-being, partly independently of physical activity (PA). The purpose of the present study was to describe trends of SB among school-aged children, adolescents, and adults in Finnish population-based studies.

**Methods:**

The study is based on three cross-sectional population-based studies: Finnish School-aged PA (FSPA) study (2020 and 2022) for 7-20-year-olds, the FinFit 2021 study for 20-69-year-olds, and the Older Adults PA study (2017-2019) for 70+-year-olds. In each study participants’ physical behavior was measured by a tri-axial accelerometer 24/7 (UKK RM42, UKK Terveyspalvelut Oy, Tampere, Finland). During waking hours accelerometer was worn on an elastic hip-band and during time in bed, a rough proxy of sleep, on a wrist-band attached to a non-dominant wrist. Parameters of SB (sitting and laying down) and standing were based on the angle for posture estimation (APE) method and analyzed in terms of total daily times, different bout lengths, and patterns of SB during the day.

**Results:**

SB covered the majority of waking hours in each participating group. The 7-year-olds had the smallest amount (on average 6h) and the 80-year-olds and older had the highest amount (over 11h) of daily SB. The amount of SB increased from younger to older participants during schoolyears, plateaued during working life, and increased again after retirement. Among children, most of the daily SB accumulated from less than 20-minute bouts, and the differences between the age groups were small. Among adults, these short bouts covered less than half of the total SB time. Bouts lasting 20-60 minutes or more than 60 minutes at a time were most frequent among the oldest participants.

**Conclusion:**

SB is a prevalent behavior in the Finnish population, regardless of age. Since physical behavior, more precisely SB, standing, light, moderate and vigorous PA, and time in bed, form a continuum for 24 hours, an increase in one behavior decreases one or more of the others. Thus, the high prevalence of SB offers great potential for changing physical behavior patterns in a more active and healthier direction.

